# Genetic diversity and structure of a recent fish invasion: Tench (*Tinca tinca*) in eastern North America

**DOI:** 10.1111/eva.13520

**Published:** 2022-12-20

**Authors:** Thaïs A. Bernos, Sunčica Avlijaš, Jaclyn Hill, Olivier Morissette, Anthony Ricciardi, Nicholas E. Mandrak, Kenneth M. Jeffries

**Affiliations:** ^1^ Department of Ecology and Evolutionary Biology University of Toronto Toronto Ontario Canada; ^2^ Department of Biological Sciences University of Toronto Scarborough Scarborough Ontario Canada; ^3^ Redpath Museum McGill University Montreal Québec Canada; ^4^ Department of Biology McGill University Montreal Québec Canada; ^5^ Maurice Lamontagne Institute Fisheries and Oceans Canada Mont‐Joli Québec Canada; ^6^ Département des Sciences Fondamentales Université du Québec à Chicoutimi Chicoutimi Québec Canada; ^7^ Department of Biological Sciences University of Manitoba Winnipeg Manitoba Canada

**Keywords:** biological invasions, bottleneck, colonization, dispersal, fisheries management, landscape connectivity, non‐native species, range expansion

## Abstract

Introduced and geographically expanding populations experience similar eco‐evolutionary challenges, including founder events, genetic bottlenecks, and novel environments. Theory predicts that reduced genetic diversity resulting from such phenomena limits the success of introduced populations. Using 1900 SNPs obtained from restriction‐site‐associated DNA sequencing, we evaluated hypotheses related to the invasion history and connectivity of an invasive population of Tench (*Tinca tinca*), a Eurasian freshwater fish that has been expanding geographically in eastern North America for three decades. Consistent with the reported history of a single introduction event, our findings suggest that multiple introductions from distinct genetic sources are unlikely as Tench had a small effective population size (~114 [95% CI = 106–123] individuals), no strong population subdivision across time and space, and evidence of a recent genetic bottleneck. The large genetic neighbourhood size (220 km) and weak within‐population genetic substructure suggested high connectivity across the invaded range, despite the relatively large area occupied. There was some evidence for a small decay in genetic diversity as the species expanded northward, but not southward, into new habitats. As eradicating the species within a ~112 km radius would be necessary to prevent recolonization, eradicating Tench is likely not feasible at watershed—and possibly local—scales. Management should instead focus on reducing abundance in priority conservation areas to mitigate adverse impacts. Our study indicates that introduced populations can thrive and exhibit relatively high levels of genetic diversity despite severe bottlenecks (<1.5% of the ancestral effective population size) and suggests that landscape heterogeneity and population demographics can generate variability in spatial patterns of genetic diversity within a single range expansion.

## 1 INTRODUCTION

Biological invasions and range expansions entail changes in population size across space and time. Following introduction to a new area, founding individuals typically carry only a fraction of the alleles present in their source population. This loss of genetic diversity, known as a genetic bottleneck, can increase inbreeding, reduce heterozygosity, and lessen the ability of introduced populations to adapt to novel environments (Chakraborty & Nei, 1977; Nei et al., 1975). Furthermore, when populations spread from restricted areas to larger regions, the number of individuals initially colonizing new habitats is likely to be limited; as a consequence, bottlenecks can occur sequentially on expanding margins (Peter & Slatkin, 2013, 2015). These serial founder events are believed to reduce the genetic diversity and the fitness of populations across space, thereby hindering their geographic spread into suitable habitat (Peischl et al., 2013; Peischl & Excoffier, 2015). Yet, rather than suffering the fate of many small populations—that is dwindling abundance—some introduced populations expand geographically and demographically (Dlugosch & Parker, 2008a, 2008b; Uller & Leimu, 2011).

Significant genetic bottlenecks are expected in populations resulting from the single introduction of a relatively small number of individuals, whereas when the number of founder individuals is large, introduced populations can retain the genetic diversity of the source population (Kanuch et al., 2021; Michaelides et al., 2016). Multiple introductions (spatial or temporal) are also common, although poorly documented, and can increase genetic diversity and population structure by adding individuals and introducing new genetic variants (Dlugosch & Parker, 2008a; Roman & Darling, 2007; Snyder & Stepien, 2017; Uller & Leimu, 2011). For many documented invasions, the consequences of bottlenecks on genetic diversity are often modest and do not hinder establishment or geographic expansion (Estoup et al., 2016). This is because invasions are often the result of large propagule sizes and multiple introductions, a pattern often associated with invasion success (Blackburn et al., 2015; Roman & Darling, 2007).

During geographic expansions, the loss of genetic diversity from the core to the margin of the expanding range is mediated by dispersal (Ibrahim et al., 1996; Waters et al., 2013). In theory, genetic diversity losses should be exacerbated in less mobile species, owing to repetitive breeding between a limited number of lineages at the expanding margin (Hallatschek et al., 2007; Hallatschek & Nelson, 2008). In addition, when long‐distance dispersal events are rare, marginal populations descending from a small number of founders are likely to suffer from the genetic consequences of bottlenecks (Gandhi et al., 2016). Alternatively, highly mobile species can retain more genetic diversity because dispersal within the expanded range will contribute genetic diversity to marginal populations (Birzu et al., 2019; Goodsman et al., 2014). In natural populations, genetic interconnectedness is also constrained by the distribution of suitable habitat and the presence of dispersal barriers (Balkenhol et al., 2015). While often investigated using simulations and mathematical models (Andrade‐Restrepo et al., 2019; Klopfstein et al., 2006; Peter & Slatkin, 2015), the outcomes of ongoing range expansion on spatial patterns of genetic diversity are not yet fully resolved empirically.

**FIGURE 1:**
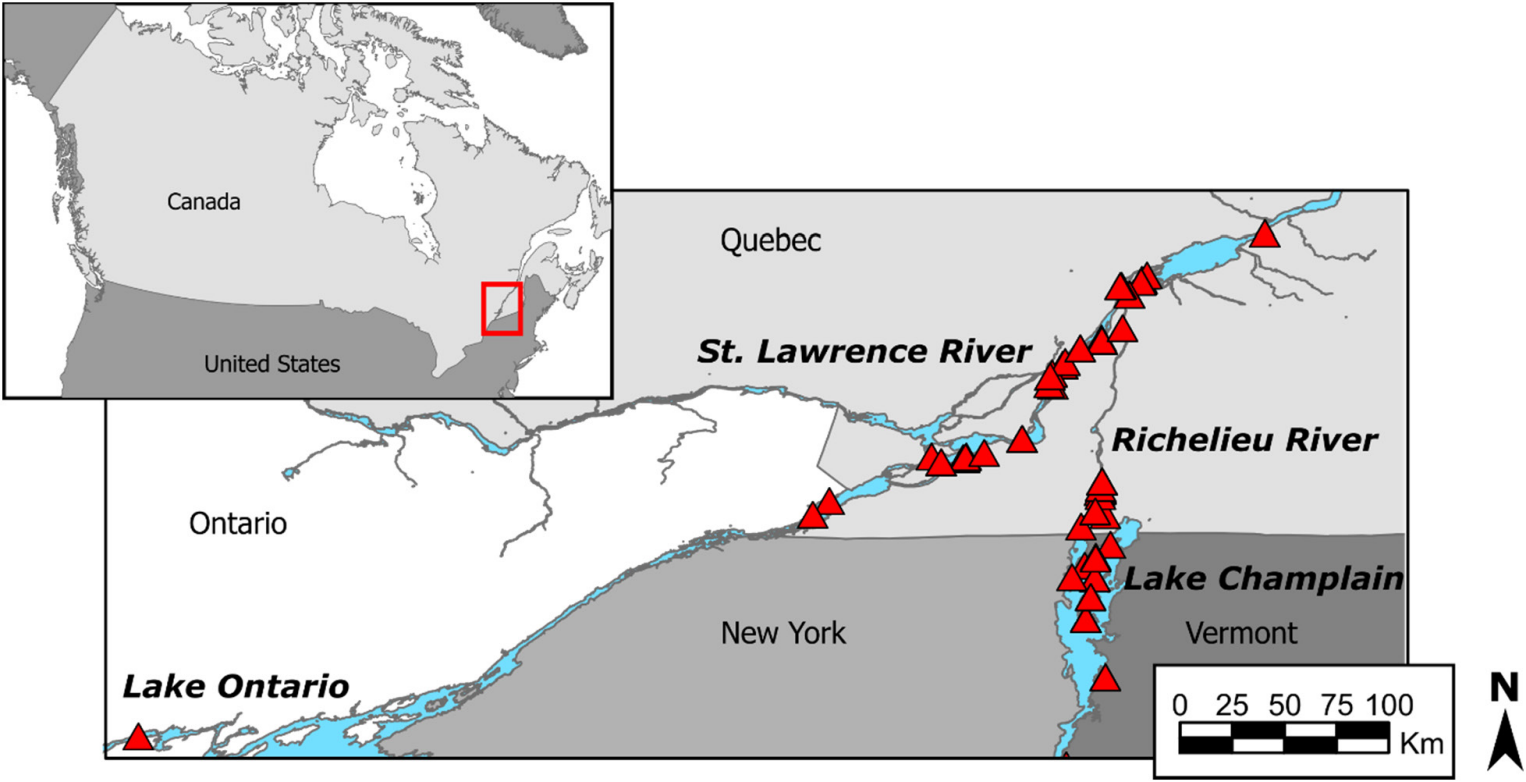
The focal area in eastern North America and locations of samples (red triangles) of the contemporary population included in the genetic analysis of Tench (*Tinca tinca*).

In parts of its native Eurasian range, the Tench (*Tinca tinca*) is a fish of conservation concern; on other continents, it is an invasive species (Avlijas et al., 2018). In eastern North America, approximately 30 individuals sourced from Germany were imported by a farmer for aquaculture purposes (Dumont et al., 2002). Initially contained into one or more farm ponds, Tench likely escaped into a network of agricultural streams flowing into the Richelieu River during the drainage of the ponds sometime between the late 1980 s and early 1990 s (Dumont et al., 2002) (Figure 1). After an initial lag period, the Tench population began growing and spreading detectably. It was first reported in Lake Champlain in the early 2000 s, it subsequently dispersed towards southern Lake Champlain (southern front) and through 500 km of riverine habitat in the St. Lawrence River (hereafter, the SLR) between Lake Ontario (western front) and Quebec City (north‐eastern front) (Avlijas et al., 2018). Multiple introductions, which are often poorly documented, could not be ruled out based on the invasion history record, as individuals of unknown origin have also been found in ponds in Quebec and in the Laurentian Great Lakes watershed (Avlijas et al., 2018). Additionally, the assumption that the Tench population in eastern North America resulted from a single introduction involving a handful of individuals has not yet been validated genetically.

To date, the Tench population in eastern North America has been expanding without active management (Avlijas et al., 2018). Detailed knowledge of levels of connectivity could inform decisions regarding interventions to manage the invasion. For example, if genomic data suggest low connectivity and patches of genetically similar individuals across the invaded range, targeted culling of individuals in patches at the margins of the invasion might limit the expansion. Conversely, if there is widespread connectivity within the invaded region, eradication might not be feasible, and thus, managers might focus their efforts on preventing Tench dispersal to, or reducing population density in areas of special concern (e.g. wetlands, spawning beds) (Gandhi et al., 2016; Low et al., 2018). Finally, owing to uncertainties surrounding the levels of connectivity within the eastern North American population, Tench's ability to disperse to new areas remains unknown.

We tested two contrasting hypotheses to characterize the invasion history of Tench in the region. The “bottleneck hypothesis” posits that the population recently underwent a drastic change in population size, presumably upon its introduction to eastern Canada. Alternatively, the “multiple‐introduction hypothesis” posits that the establishment and geographic expansion of Tench resulted from more than one release event from distinct genetic sources. These scenarios are characterized by contrasting levels of genetic diversity and population structure across time and space, as well as evidence, or lack thereof, for a recent bottleneck. Following conventional genomic analyses based on clustering and metrics of genetic differentiation using SNPs from restriction‐site‐associated DNA sequencing, we computed individual‐ and population‐based metrics of genetic diversity. To test for the occurrence of a recent bottleneck, we matched our empirical data to scenarios of bottlenecks generated using coalescent‐based simulations.

We also employed powerful spatially informed population genomic approaches to test two hypotheses concerning the connectivity of the population within the invaded region. Under the “low‐connectivity hypothesis,” individuals do not typically disperse over long geographic distances. If spread is constrained by landscape heterogeneity or dispersal capacity, the population might exhibit local patches of genetically similar individuals and small genetic neighbourhoods (the area within which the impact of gene flow on genetic diversity is higher than that of drift, so that the area is close to panmictic) (Wright, 1946). Alternatively, under the “high‐connectivity hypothesis,” where Tench are capable of large‐scale movements (>100 km) within the established range, effective gene flow across large distances will result in a genetically cohesive population unit across the invaded range and large genetic neighbourhood size. Finally, genetic diversity metrics should vary as a function of connectivity. Specifically, if local numbers of breeding individuals are small due to their relative isolation, which increases with geographic or ecological distances, genetic diversity metrics will vary across the invaded range. The eastern North American Tench population presents an opportunity to examine the patterns of genetic diversity resulting from the introduction and geographic spread of a riverine fish, especially if this successful invasion resulted from a small initial population as the record of its introduction suggests.

## 2 MATERIALS AND METHODS

### 2.1 Data

#### 2.1.1 Study system and sampling

Tench were sampled across their eastern North American range (Figure 1), spanning southeastern Canada (SLR, Richelieu River, and one sample from Lake Ontario) and northern Vermont, U.S. (Lake Champlain). They were captured using a variety of methods (i.e. electrofishing, gillnets, fyke nets, and seine nets) from 2016 to 2019. The captured individuals were geolocated and sampled for genetic analyses; tissue samples were preserved in 95% ethanol and held in −25°C freezers until extraction. DNA was extracted using Qiagen DNA extraction kits (Qiagen). To capture spatial patterns of genetic variation across the invaded range at finer geographic scales, we extracted DNA from a total of 345 samples collected throughout the known invaded range and quantified extracted samples with a Qubit (Thermo Fisher). We then sequenced those with DNA concentrations greater than 10 ng/μl (*n* = 238). To further understand the demographic history of the population, we also extracted DNA from 40 archived fin clips collected from individuals sampled in 2002, when the species was still geographically restricted to the Richelieu River. While the DNA was degraded for most of those fin clips, we were able to include 10 samples (hereafter referred to as the historical samples) in the sequencing effort.

#### 2.1.2 Genomic data

Restriction‐site‐associated DNA (RAD) libraries were prepared following a three‐enzyme protocol (Bayona‐Vásquez et al., 2019), with modifications described in detail elsewhere (Lujan et al., 2020). Briefly, the enzymes XbaI and EcoRI were used to digest the genome and NheI to separate adapter‐dimers. Isolated fragments were 340–450 bp long, ensuring that the sequencing reads (61 bp) would be at least 280 bp from all other loci, thereby helping meet the assumption of unlinked loci for downstream analyses. Libraries were sequenced on the Illumina NextSeq500 Desktop sequencer v2 for 75 bp single‐end reads (Illumina Inc.) at the University of Toronto Center of Analysis of Genome Evolution and Function (CAGEF). We demultiplexed the resulting sequences using bcl2fastq2 v2.20 (Illumina). We then discarded low‐quality reads and trimmed reads to 61 by removing heterogeneity spacers, restriction overhangs, and compensatory bases, using fastp v0.20 (Chen et al., 2018). We visualized sequence quality with FastQC v0.11.8 (Andrews, 2010) and multiQC v1.9 (Ewels et al., 2016).

Next, we filtered raw reads, assembled them de novo, and identified variants using the stack v2.3 pipeline (Catchen et al., 2013). For parameter optimization, we ran STACKS several times on the entire dataset, varying the values for the ustack parameter M (the number of mismatches allowed between stacks to merge them into a putative locus) from 1–5 (M1‐M5) and the cstack parameter *n* (the number of mismatches allowed during the construction of the catalog) from M − 1 to M + 1. The other parameters were kept constant as they were shown to work well in previous reviews of stacks' parameter space (Paris et al., 2017; Rochette & Catchen, 2017) and were as follows: process_radtags (−‐clean, −‐quality, −‐filter_illumina, −t 61, −‐disable_rad_check); ustacks (−‐disable‐gapped, −‐model_type bounded, −‐bound_high 0.05, −max_locus_stacks 4, −m 3, ‐H); cstacks (−‐disable‐gapped); and, population (−R 0.70, min‐mac 2, −‐vcf). For each run, we visualized the effect of M and n values on several metrics, including the number of loci and polymorphic loci shared by 70% of the samples, the distribution of single nucleotide polymorphisms (SNPs) per loci, and the proportion of loci with excess heterozygosity (H > 0.55) or a read ratio deviation (D) greater than 7 inferred with HDplot (see below). Based on the effect of M and n values on these metrics (Figure S1), we identified M2 and n2 as optimal parameters as they maximized polymorphism while minimizing the number of potentially erroneous SNPs.

The resulting data were filtered with vcftools v1.16 (Danecek et al., 2011) for data missingness. Specifically, we started with low cut‐off values for missing data applied separately per individual and locus that we then iteratively and alternatively increased to exclude low‐quality locus and individuals (Leary et al., 2018). Final filters included sites genotyped at >70% of individuals, individuals genotyped at >70% of loci, and polymorphic loci with a minor allele count greater than two. For the missingness filters, we chose these threshold values as they maximized the number of individuals and loci retained for downstream analyses while ensuring the quality of the data. We imported the resulting data into HDplot (McKinney et al., 2017) to investigate allelic read depth and heterozygosity; we removed SNPs with an H greater than 0.55 or |D| > 7 as they could result from potential error in loci splitting and bioinformatics. Finally, in loci with multiple SNPs, we selected the SNP with the highest minor allele frequency for downstream analyses. As no individuals related at the level of full or half‐sibling (mean [range] r = 0.01 [0–0.20]) were detected using the maximum‐likelihood estimation of pairwise relatedness implemented in SNPRelate (Zheng et al., 2012), no clustering patterns were closely associated with three pairs of individuals with higher relatedness (>0.15) alone, and all individuals were kept in the dataset.

### 2.2 Invasion history analysis

#### 2.2.1 Bottleneck evaluation

**FIGURE 2:**
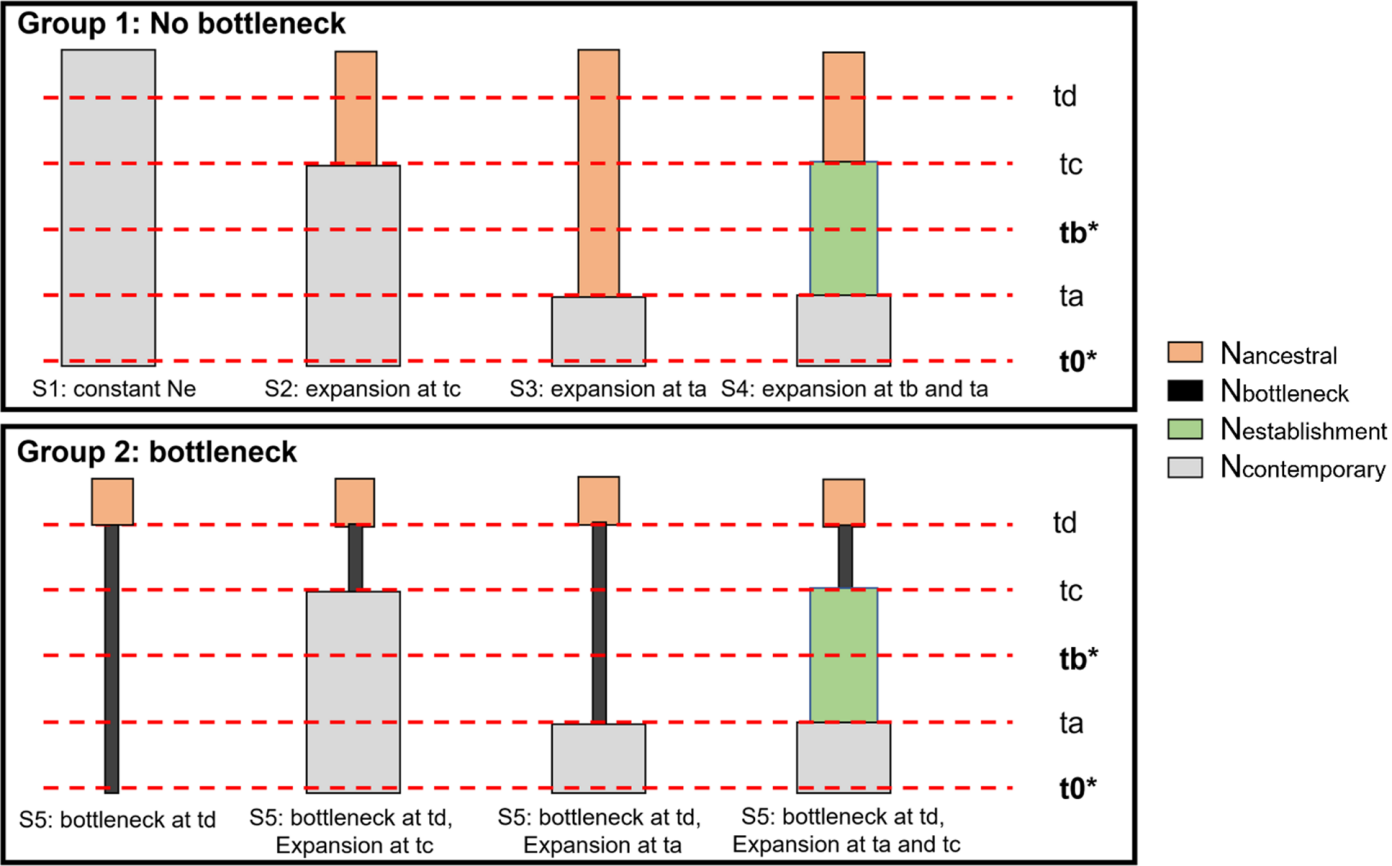
Schematic representation of the two groups of scenarios for the introduction history of Tench (*Tinca tinca*) in eastern North America tested with DIYABC Random Forest. N represents the effective population size in the ancestral, bottleneck, established, and contemporary population; t represents timing events, including sampling events (in bold*: t0 = contemporary, tb = historical population), population expansion between the contemporary and the historical population (ta) and between the bottleneck and the historical population (tc), and population bottleneck (td in group 2 of scenarios).

To test whether the Tench population experienced a genetic bottleneck, we used the approximate Bayesian Computation random‐forest method in DIYABC Random Forest v1.0 (Collin et al., 2021). We simulated training sets under two groups of competing scenarios referring to the absence (group 1: scenarios 1, 2, 3, and 4) or the occurrence (group 2: scenarios 5, 6, 7, and 8) of a recent bottleneck (Figure 2). Demographic parameters included four population sizes (Nancestral, Nbottleneck, Nestablishment, and Ncontemporary), two sampling events t0 (for the contemporary samples) and tb (for the historical samples), and up to three changes in effective population sizes (ta for changes between the contemporary and the historical samples, tc for changes before the historical samples, and td for changes between the ancestral and the bottleneck population). Prior values were drawn from uniform distributions with t0 < ta < tb < tc < td (going backwards in time). We parameterized the bottleneck timing and population size (td = [1–100]; Nbottleneck = [2–100]) to reflect the reported importation of about 30 Tench specimens to Quebec in the 1980s (Dumont et al., 2002). Sampling events reflected the sampling of the contemporary (t0 = [0–5]) and historical (tb = [2–15]) population in 2017–19 and 2002, respectively. We used uniform prior values between 2 and 15 to reflect population expansion between the contemporary and the historical samples (ta) and between 2 and 50 to reflect population expansion before sampling of the historical population (tc). Finally, we used a broad range of priors to reflect uncertainties around population sizes (Nancestral, Ncontemporary, Nestablishment = [2–10,000]). Under all scenarios, we assumed that the population was the result of a single introduction (see **Results**).

To identify the most likely demographic trajectory, we conducted the scenario‐choice analysis twice. First, we tested whether the occurrence of a genetic bottleneck was important by conducting an analysis at the group level (with vs. without bottleneck). Second, we considered the eight scenarios separately. The training set included a total of 40,000 simulated datasets (i.e. 5000 per scenario), and we fixed the number of trees in the constructed random forest to 1000. Next, for scenario 5 (the best‐suited scenario among the set of 8: see **Results**), we estimated the parameters involved in the invasion history: ancestral (N_ancestral_) and bottleneck population size (N_bottleneck_) and the bottleneck time (td). For this analysis, the training set included 10,000 simulated datasets and 1000 trees in the random forests. We ensured that the number of simulated datasets was sufficient for scenario selection and parameter estimation by evaluating the stability of both results and accuracy metrics (results not shown).

#### 2.2.2 Temporal changes in genetic diversity and effective population size

We computed several measures of genetic diversity for both historical and contemporary populations. At the population level, we calculated observed heterozygosity (Ho), within‐population gene diversity (Hs), and within‐population inbreeding coefficient (Fis) using HierFstat (De Meeus & Goudet, 2007). These metrics are either insensitive to (Ho, Fis) or corrected for (Hs) sample sizes. We also computed two metrics of genetic diversity at the individual level: multilocus heterozygosity (MLH), defined as the number of heterozygous SNPs divided by the number of SNPs genotyped, was calculated using the package inbreedR (Stoffel et al., 2016), and Individual Relatedness (IR), a metric related to the relative location of individuals along the outbred‐inbred continuum, using the package Rhh (Alho & Valimaki, 2012). Negative IR values indicate outbreeding, positive values inbreeding. We tested for temporal changes between the historical and contemporary populations in samples' Ho and Hs using Wilcoxon sign‐ranked test with locus treated as paired measures between populations. Because the number of samples was highly uneven between the historical and contemporary populations (biased towards the contemporary population), we used randomization tests with 5000 samples to produce accurate estimates of the *p* values for temporal differences in average MLH and IR.

To provide further insights into temporal changes in eco‐evolutionary dynamics, we also estimated the effective population size using the linkage disequilibrium (Ne_LD_) method implemented in NeEstimator V2.1 (Do et al., 2014). This method assumes that, in small populations, heightened drift causes nonrandom associations between unlinked alleles. For our sample size, the least biased estimates (i.e. excluding singleton alleles) is for allele exclusion criteria Pcrit = 0.1; however, we evaluated how rare alleles affected Ne by looking at Ne variation across the range of Pcrit value (0, 0.1, 0.2, and 0.5). Stable Ne across Pcrit values are suggestive of stable, isolated populations, whereas high variance across Pcrit values could highlight demographic processes resulting in excess of rare alleles, such as contemporary gene flow (Waples & England, 2011) or demographic expansion (Excoffier et al., 2009). We do not discuss the results from the linkage disequilibrium method in the historical population as it produced infinite estimates, a likely consequence of the small sample size relative to the effective population size.

#### 2.2.3 Spatiotemporal patterns in genetic structuring

To identify potential clusters of genetically differentiated populations across time and space, we used two nonspatial analytical approaches: a principal component analysis (PCA) to explore patterns in the genetic data and a discriminant analysis of principal component (DAPC) using the R package Adegenet (Jombart & Ahmed, 2011) to identify clusters of genetically similar individuals. In these analyses, historical and contemporary samples were included together. We evaluated the degree of population differentiation based on Fst and associated bootstrap confidence intervals. PCA creates synthetic variables to maximize variation among samples. In contrast, DAPC identifies groups of genetically similar individuals by transforming the raw data using PCA and then performing a discriminant analysis on the retained PCs to maximize between‐group variability while neglecting within‐group variation (Jombart et al., 2010). We used DAPC without the a priori assumption of population structure. To assign samples to groups subsequently used as population identifier, we performed K‐mean clustering from K1 to K10 with all PCs retained. To identify the best K, we used the “diffNgroup” criterion, which identifies the best K based on Bayesian Information Criterion (BIC) differences between successive values of K, as well as the “min” criterion, which retains the model with the smallest BIC. We determined how many PCs to retain based on cross‐validation: DAPC were performed on a training dataset comprising 90% of the samples in each subpopulation with different numbers of PCs retained and then used this to predict the group of the remaining 10%. We retained the number of PCs associated with the lower mean squared error to perform the DAPCs.

### 2.3 Fine‐scale population genomics and dispersal

#### 2.3.1 Within‐population subdivision

To assess contemporary population subdivision within the invaded range and identify potential areas of spatial discontinuities, we used multivariate methods that integrate spatial information into the analyses of genetic dissimilarity (i.e. spatial principal component analysis (sPCA), MEMgene). For these analyses, the historical samples were excluded. sPCA aims to identify spatial genetic patterns by analysing spatial autocorrelation. To do so, it computes a matrix of Moran's index inferred from the comparison between individual allelic frequencies to that of a user‐defined connection network (Jombart et al., 2008). Variation is then analysed with respect to variables (eigenfunctions) representing geographic variation that are attributed to positive (when individuals in the same neighbourhood have similar allelic frequencies, referred to as global structure) or negative (when individuals in the same neighbourhood have dissimilar allelic frequencies, referred to as local structure) autocorrelations. We set the connection network to a minimum distance neighbour graph. To characterize distance between samples, we first computed the shortest river network distance between samples that we then re‐projected as Cartesian coordinates using nonmetric multidimensional scaling (nMDS) in the vegan package (Oksanen et al., 2019). We then tested for significant global and local structure using the Monte Carlo simulation with 999 permutations. For visualization, we retained the two largest positive values and the three largest negative values. We performed the sPCA in Adegenet (Jombart & Ahmed, 2011).

MEMgene uses Moran's eigenvector maps to analyse a weighted connection network. The identified spatial patterns, known as MEM eigenvectors, describe the patterns of positive and negative autocorrelation in the data (Galpern et al., 2014). The analysis then implements a forward selection procedure to identify the MEM eigenvectors that are statistically significant in a genetic distance matrix. For this reason, it performs better than sPCA in fragmented landscapes and highly mobile organisms (Galpern et al., 2014). In this analysis, we used least‐cost river network distance between samples as weights in the network and the proportion of shared alleles as genetic distance. We implemented the forward selection of the statistically significant MEM eigenvectors to identify spatial patterns and used R^2^adj to estimate the strength of these spatial patterns.

### 2.4 Genetic neighbourhood size and spatial variation in genetic diversity

To understand how genetic diversity is spatially distributed across the invaded range, we used sGD (Shirk et al., 2011) to compute metrics of genetic diversity based on the genetic neighbourhood surrounding each individual. To identify the size of a genetic neighbourhood, defined as the distance at which pairwise genetic distances are no longer significantly correlated (Wright, 1946), we produce Mantel correlograms across a range of distance classes using the ecodist package (Goslee & Urban, 2007). We identified the genetic neighbourhood as the first distance class at which spatial correlation was no longer statistically significant (Shirk et al., 2011). For this analysis, we explored distance classes from 10 km to 300 km at intervals of 10 km and ran each test with 999 permutations. Next, for a set radius of 220 km (the genetic neighbourhood size based on Mantel correlograms: see **Results**), we computed observed heterozygosity (Ho), expected heterozygosity (He), and allelic richness (Ar) for each genetic neighbourhood with a minimum sample size of 20 individuals. We did not compute these metrics for the Lake Ontario sample because no other samples were within its genetic neighbourhood distance. Finally, as a comparison to the neighbourhood grouped metrics of genetic diversity, we also examined spatial variation in the individual‐level metrics of genetic diversity (IR, MLH).

Next, we used linear regressions to examine the influence of range expansion on genetic diversity metrics. We included river distance (least‐cost distance following the watercourse) from the putative origin, individual location relative to the putative origin (north or south), and their interaction, as explanatory variables in the models. To select the best models, we conducted backwards model selection by testing the significance of the fixed effects with likelihood‐ratio tests. We started with the interaction term and removed nonsignificant fixed effects from the models. We removed the Lake Ontario individual whose distance from the putative origin of the invasion was 4.5‐fold greater than the mean distance and, consequently, had a disproportionate influence on the linear regressions; however, interpretation of the results was consistent across analyses including or excluding this individual. We used gdistance (Van Etten, 2017) to compute the river distance between each of the samples and the putative site of introduction in the Richelieu River.

## 3 RESULTS

### 3.1 Genomic data

We identified 8300 SNPs in the full Tench dataset; of those, only 28% were polymorphic. After filtering, 1898 SNPs for 203 individuals remained in the final dataset (195 contemporary samples and 8 historical samples). Many of the individuals discarded from the analysis were from dried fin clips, and the loss of those individuals is likely due to degraded DNA. The average read number per individual was 2,401,426 (SD = 998,292). Overall, missing data averaged 11.9% (SD = 7.8%) per individual and 11.9% (SD = 5.9%) per loci across the entire dataset. Average individual missingness was 20.1% (SD = 5.7%) and individual depth 100 (SD = 9.6) for the historical samples; average individual missingness was 11.6% (SD = 7.7%) and depth 167 (SD = 58) for the contemporary samples.

### 3.2 Demographic history

The DIYABC Random Forest analysis showed clear evidence of a recent bottleneck in the SLR‐Richelieu‐Champlain Tench population. Classification votes and estimated posterior probabilities were supportive of scenario group 2 (1000 votes out of the 1000 RF trees, posterior probability = 0.996), which included the contemporary bottleneck. When considering the eight scenarios independently, the highest classification votes and estimated posterior probabilities were for scenario 5 (820 votes out of the 1000 RF trees, posterior probability = 0.832), which assumed a contemporary population bottleneck and no subsequent recovery. We observed that our capacity to discriminate between scenarios was better at the group than the scenario level, as shown by the lower overlap between the simulated datasets on the LD axes (Figure S4) and the lower global prior rate (0.029 and 0.483, respectively).

Focusing on scenario 5, parameter values had broad confidence intervals spanning most of the prior range. Estimated parameters (median [95% CI]) were as follows: 5244 [2138–9375] for the ancestral population size, 75 [37–99] for the bottleneck population size, and 52 generations [25–93] for the timing of the bottleneck event. Estimations were substantially more accurate for the bottlenecked population size and its timing than the ancestral population size, as determined by lower normalized mean absolute error (NMAE) values (results not shown).

### 3.3 Temporal changes in genetic diversity and effective population size

**FIGURE 3:**
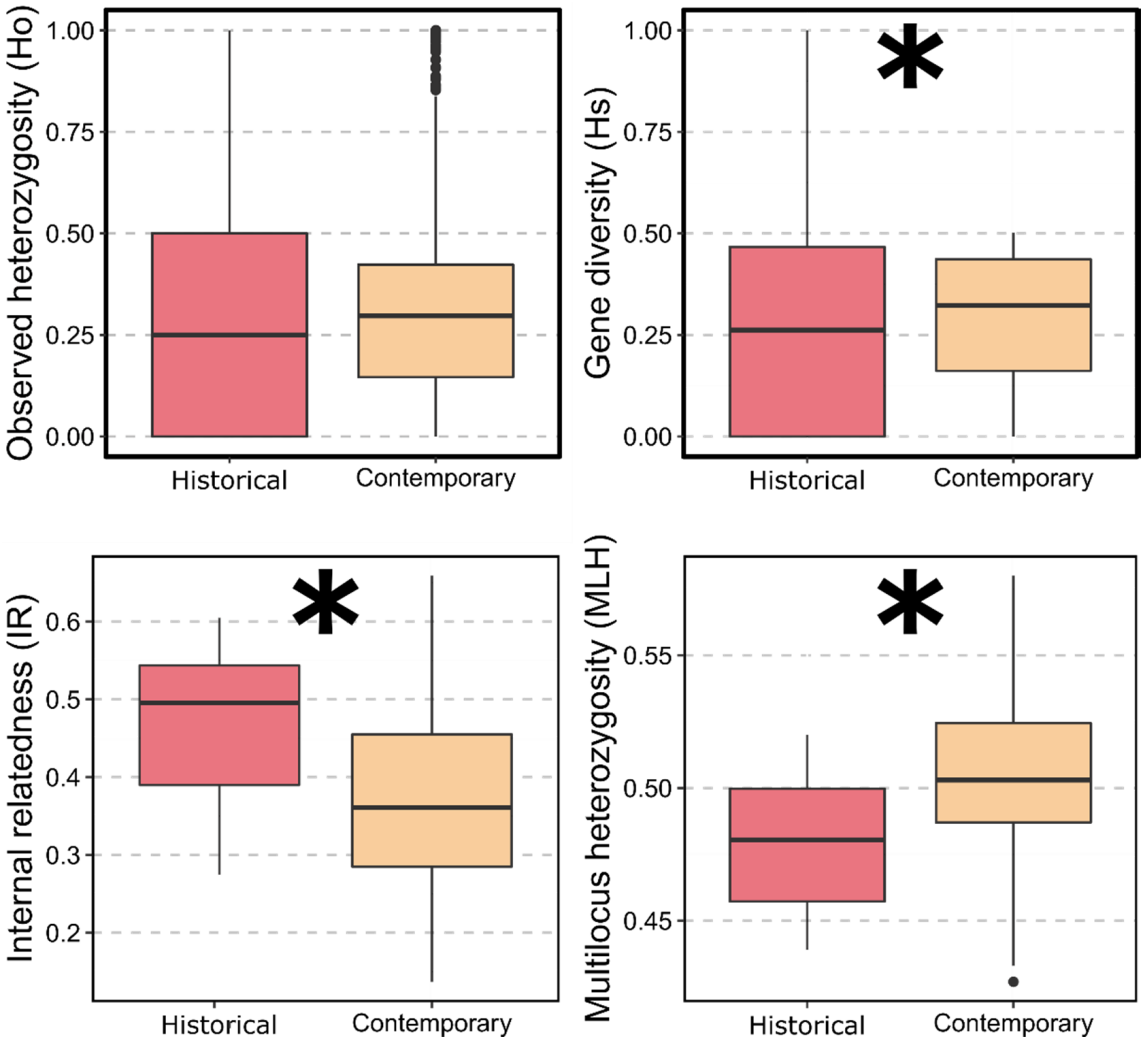
Temporal changes in genetic diversity of Tench (*Tinca tinca*) in eastern North America at the population (Ho, Hs) and the individual level (IR, MLH). The asterisks highlight significant differences (*p* < 0.05) in average metric values between the historical and the contemporary samples. IR, Individual Relatedness; MLH, multilocus heterozygosity.

At the population level, there was no statistically significant difference (*p* = 0.461) in observed heterozygosity between the historical (Ho mean [SD] = 0.308 [±0.276]) and the contemporary samples (Ho = 0.298 [±0.196]) (Figure 3). In contrast, within‐population gene diversity was significantly higher (*p* < 0.001) in the contemporary (Hs = 0.295 [±0.159]) relative to the historical sample (Hs = 0.272 [±0.200]); the change represents an average gain of 2.3% of polymorphism between samples. Observed heterozygosity was higher than Hs by 3.6 and 0.3% in the historical and the contemporary samples. Accordingly, Fis was negative and significantly different from zero in the historical (95% CI = [−0.172 to 0.119]) but not in the contemporary sample ([−0.020 to 0.006]). Finally, in the contemporary sample, we obtained a point estimate for Ne_LD_ of 113.8 (95% CI = [106–122.5]). We observed that Ne_LD_ varied by nearly 25% across Pcrit values (Figure S2).

At the individual level, genetic diversity was lower in the historical sample (Figure 3, Figure S3). Historical specimens had significantly (*p* = 0.04) higher internal relatedness (mean IR [SD] = 0.468 [0.109]) than the contemporary ones (IR = 0.379 [0.116]) and significantly lower values (*p* = 0.015) of multilocus heterozygosity (mean MLH_historical_ [SD] = 0.479 [0.029], MLH_contemporary_ [SD] = 0.504 [0.0279]). These changes represent a loss of 8.9% of the inbreeding and a gain of 2.5% of polymorphism present at the individual level between the 2002 and the contemporary samples. The internal relatedness values were all positive, indicating that both samples showed signs of inbreeding.

### 3.4 Spatiotemporal patterns in genetic structuring

**FIGURE 4:**
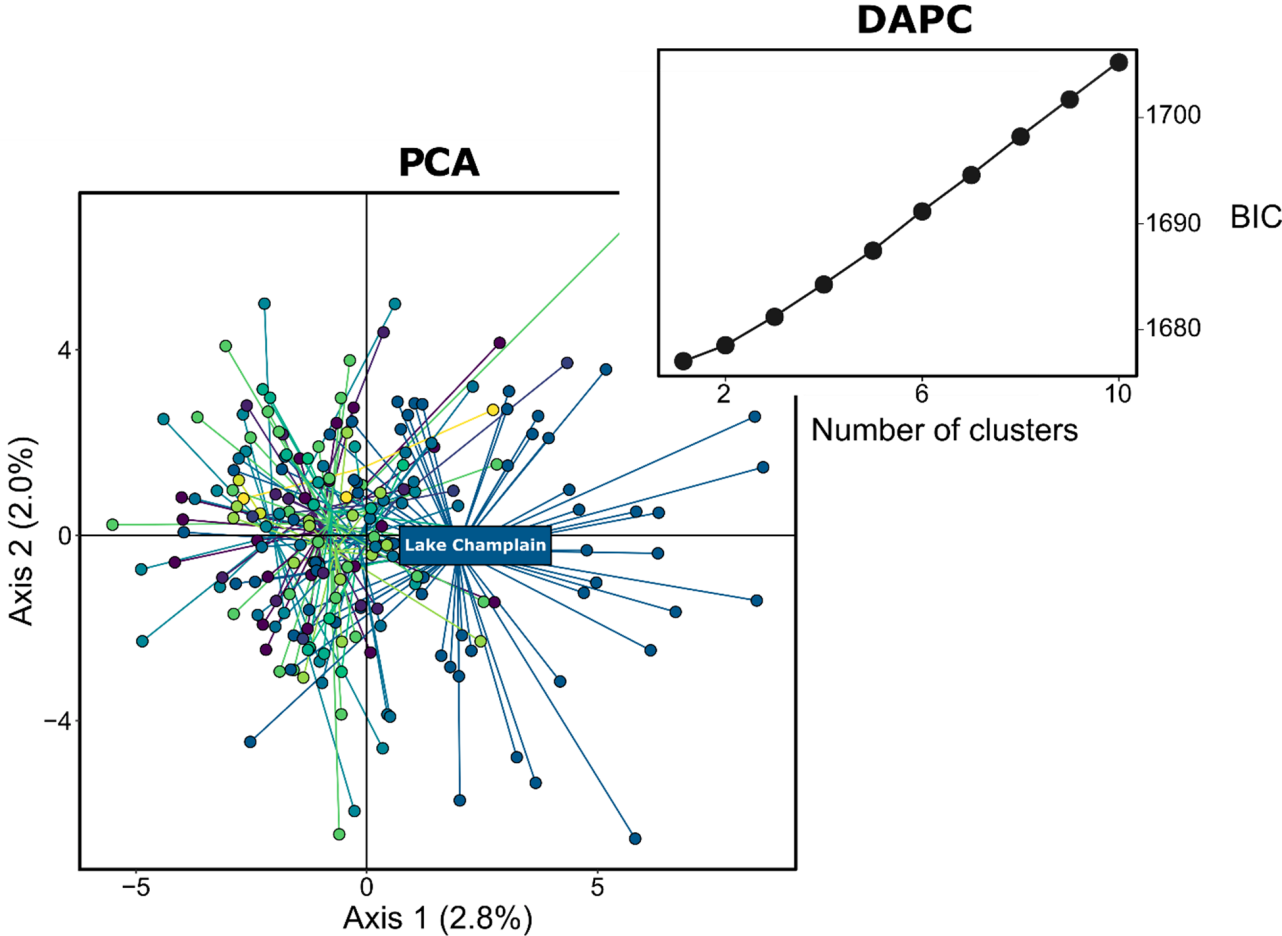
Spatiotemporal patterns in genetic structuring for Tench (*Tinca tinca*) in eastern North America were explored using several analyses. The principal component analysis showed that, with the exception of Lake Champlain that was weakly differentiated on Axis 1, the centroid of all other sampled areas (i.e. Richelieu River, Lake saint Pierre, Lake Saint Louis, Contrecoeur, Repentigny, Sorel, Montreal) and the historical samples overlapped. The discriminant analysis of principal component indicated K = 1 as the “best” number of clusters.

The PCA, DAPC, and tests of genetic differentiations indicated that the Tench population of eastern Canada, including both historical and contemporary individuals, is best described as a single genetic population. In the PCA, PC1 and PC2 cumulatively accounted for less than 5% of the total variance in genetic diversity (Figure 4). While there was no clear pattern emerging from the distribution of individuals along PC2, PC1 separated Lake Champlain Tench from the rest of the samples. However, this pattern was not resolved with the de‐novo DAPC analysis. Results of the K‐mean clustering analysis showed that K increased linearly from 1–10 (Figure 4), while the lowest BIC score was for K = 1, the greatest difference between successive BIC value was reached for K = 4. However, there were no associations between the de novo‐identified clusters and sampling locations across time and space. Results from the PCA and DAPC analyses were consistent across a range of filtering schemes, including more lenient HDplot loci filtering (e.g. H > 0.75 and |D| > 25) and data missingness threshold. Concomitantly, while Fst was significantly different from zero, its value was below 1.5% (Fst [95% bootstrapped CI] = 0.0067 [0.0049–0.0136]).

### 3.5 Fine‐scale population genomics and dispersal

**FIGURE 5:**
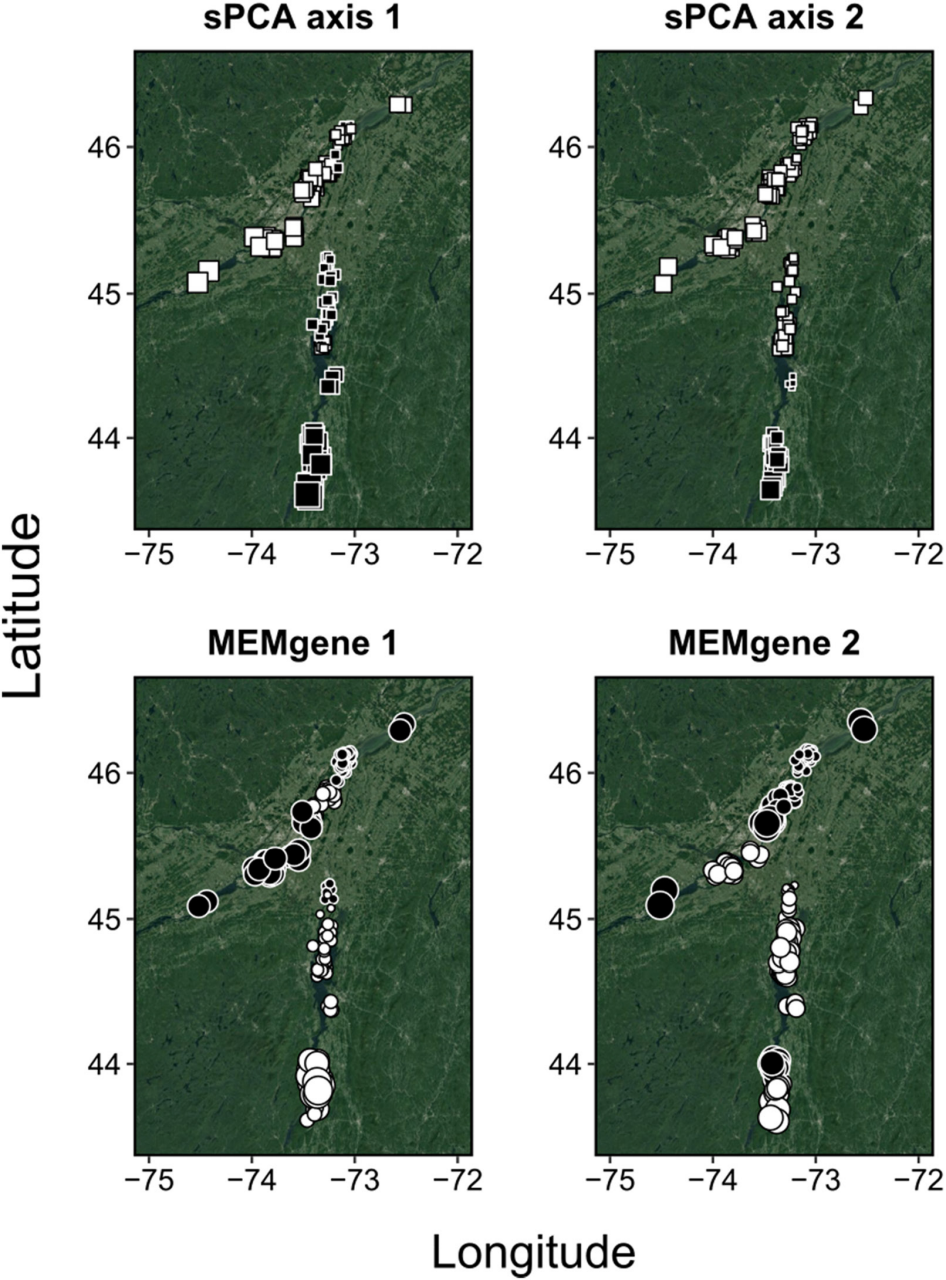
sPCA and MEMgene highlighted contrasted patterns of population sub‐structuring of Tench (*Tinca tinca*) in eastern North America. Squares (sPCA) and circles (MEMgene) represent samples. To interpret the strength of genetic differentiation, square size is proportional to the eigenvalue score and colour indicates the sign: Large white square is very much differentiated from large black square, and small squares are less differentiated. To facilitate the visual interpretation of the plots, The Lake Ontario sample was removed.

Together, the sPCA and MEMgene analysis revealed patterns of weak within‐population sub‐structuring across the contemporary range of invasive Tench. In the sPCA, the global test suggested statistically significant global structure across the invaded range (max(t) = 0.001, *p* = 0.001). For visualization, we retained the two largest positive values. sPCA axis 1 showed a clinal pattern of genetic differentiation across the invaded range (Figure 5). Genotypes located at the front of invasion showed the most extreme scores, but there were no sharp boundaries between individuals; rather, the change was progressive, with individuals located in the middle having less extreme scores. The second axis (Figure 5) captured the same cline in allelic diversity, with a subtle difference: individuals were more similar than expected by chance in the southern section of Lake Champlain. There was no significant local structure across the invaded range (*p* = 0.071). In the MEMgene analyses, a spatial pattern of genetic structuring emerged that was not evident in the sPCA analysis (Figure 5). Specifically, the spatial genetic pattern was more clustered, with circles of similar size and colour found in proximity, suggesting a more fragmented landscape. Overall, however, the strength of these spatial genetic patterns was weak (*R* ^2^ = 0.022).

### 3.6 Genetic neighbourhood size and spatial variation in genetic diversity

**FIGURE 6:**
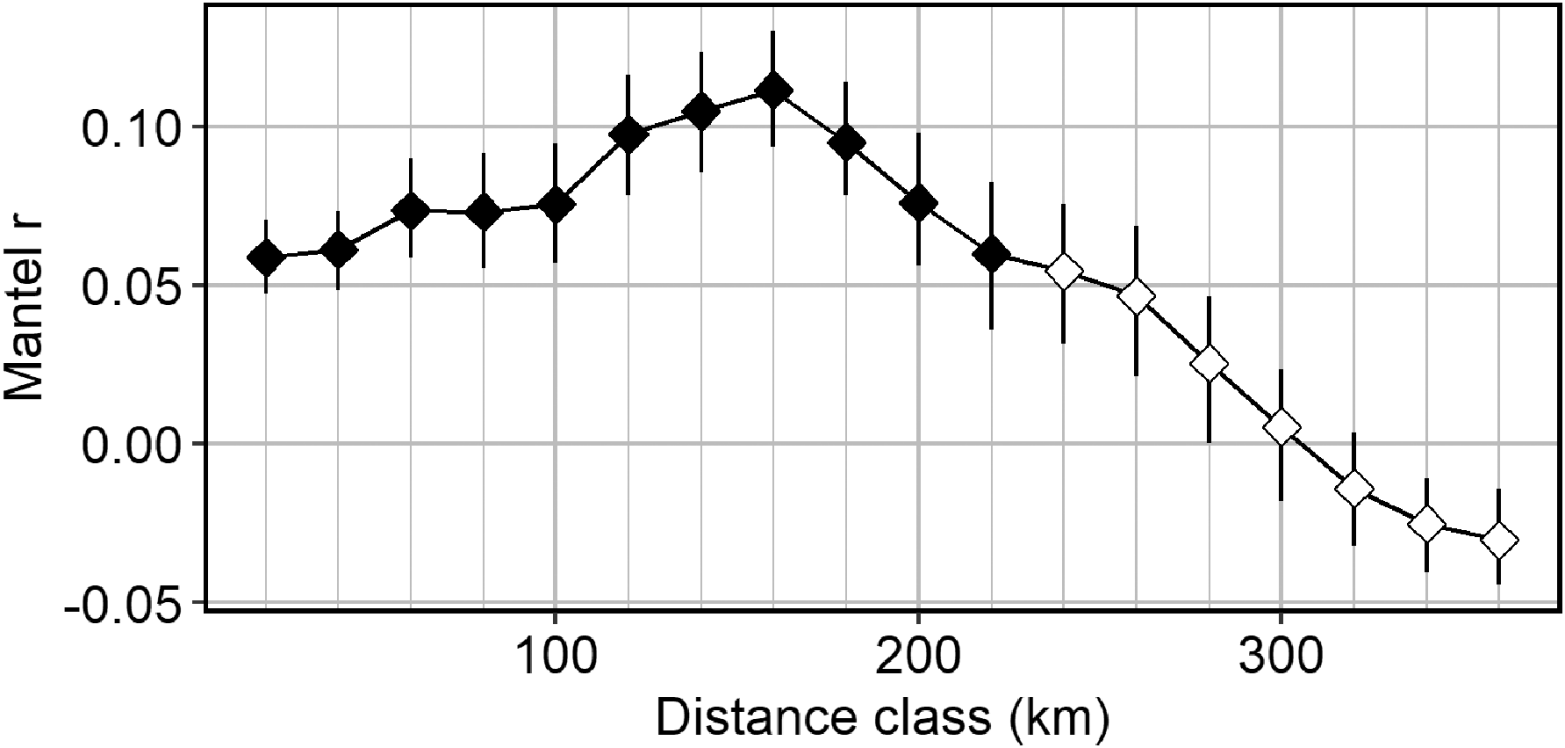
Correlogram showing spatial autocorrelation in genetic distance across a range of distance classes between Tench (*Tinca tinca*) individuals in eastern North America. The genetic neighbourhood is defined as the largest distance class with a statistically significant (indicated in black) positive correlation.

The Mantel correlograms revealed the presence of significant positive spatial structure up to the distance class of 220 km (Figure 6). Estimates of genetic diversity at the genetic neighbourhood scale did not vary widely across the invaded range; mean (min, max) estimates were 1.907 (1.904, 1.910) for Ar, 0.298 (0.295, 0.302) for Ho, and −0.011 (−0.016, −0.005) for FIS.

**FIGURE 7:**
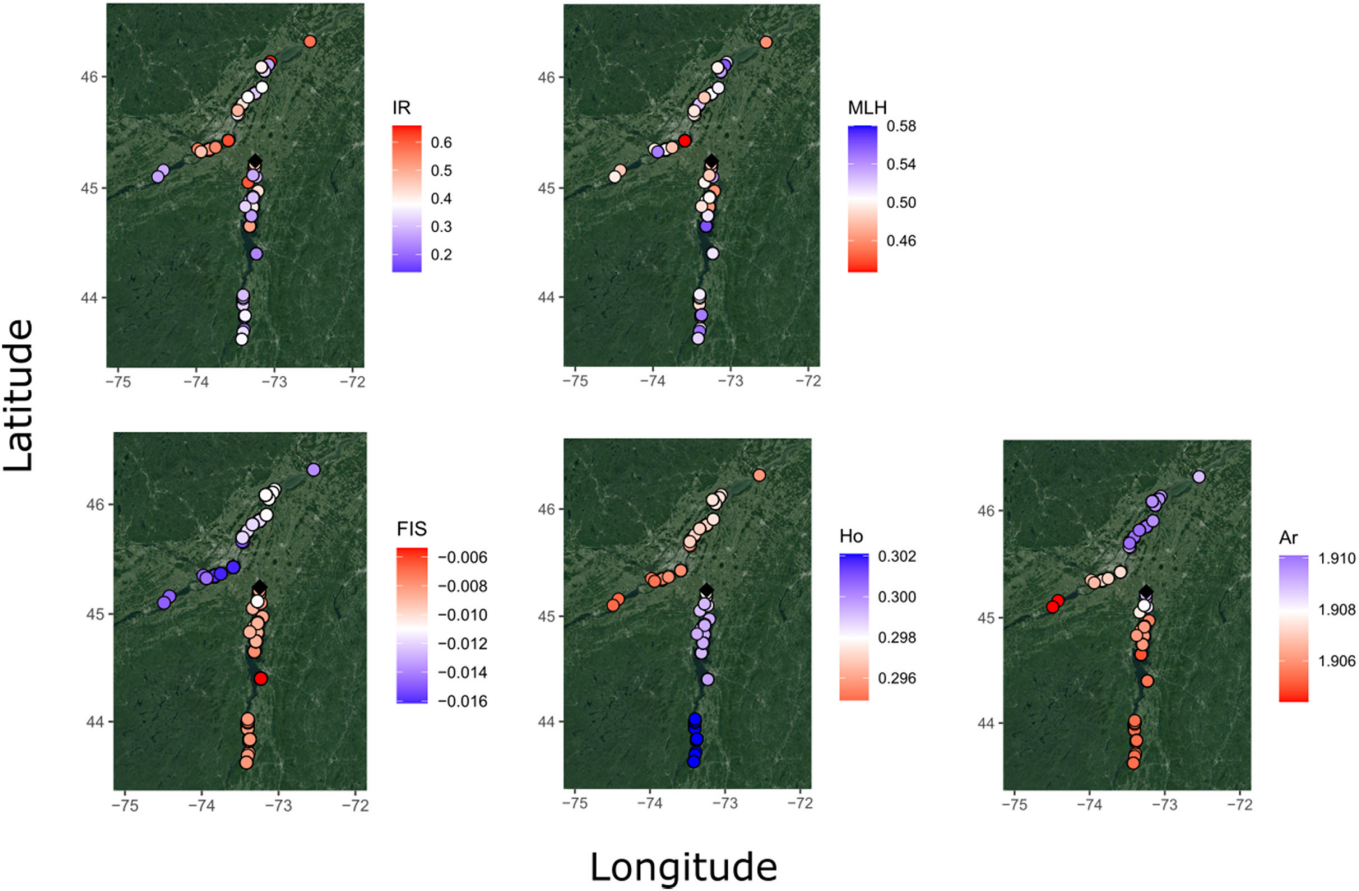
Spatial patterns of genetic diversity for the invasive population of Tench (*Tinca tinca*) in eastern North America. Genetic diversity was calculated at the individual (internal relatedness IR; multilocus heterozygosity MLH) and the genetic neighbourhood size level (inbreeding Fis; observed heterozygosity Ho; allelic richness Ar).

Linear models highlighted contrasting trends in genetic diversity metrics as a function of invasion directionality (whether individuals were on the southern or the northern side of the introduction site) (Figure 7). In the MLH model, the only significant fixed effect was distance (adj. *R* ^2^(191) = 0.02, *p* = 0.046); specifically, as distance from the origin increased, MLH decreased (−0.007 MlH/100 km). In all other models, the interaction between least‐cost river distance and individual location relative to the introduction site was significant. These models included the IR model (adj. *R* ^2^(189) = 0.095, *p* < 0.001) and, at the genetic neighbourhood level, the Ar (adj. *R* ^2^(189) = 0.85, *p* < 0.001), the Ho (adj. *R* ^2^(189) = 0.98, *p* < 0.001), and the FIS model (adj. *R* ^2^(189) = 0.92, *p* < 0.001). Ar richness decreased by 0.003 and <0.001 with distance from the origin on the northern (towards the SLR) and southern (towards Lake Champlain) invasion axes, respectively. For the other metrics (ID, Ho, and FIS), trends were in opposite directions on the two invasion axes.

In general, changes in genetic diversity metrics were of small magnitude (Figure 7). Specifically, individuals were more inbred (+0.08 IR unit/100 km) as distance from the origin increased on the northern invasion axis (towards the SLR), and less inbred (−0.06 IR unit/100 km) on the southern invasion axis (towards Lake Champlain). Genetic neighbourhoods became slightly less heterozygous (−0.002 Ho/100 km) and FIS decreased (−0.004 FIS/100 km) on the northern invasion axis. On the southern axis, heterozygosity (+0.003 Ho) and FIS increased (+0.001 FIS/100 km) with distance.

## 4 DISCUSSION

We analysed genomic data from the invasive population of Tench in eastern North America to discriminate between hypotheses related to the species' demographic history and connectivity. Using a dataset with 1898 SNPs for 203 individuals, we found low effective population size, a lack of marked population subdivision across space, and evidence of a recent genetic bottleneck. Consistent with the presumed invasion history of the species in Quebec (Avlijas et al., 2018; Dumont et al., 2002), our results confirm that multiple introductions from genetically distinct sources are unlikely and imply that introduced populations can thrive despite recent bottlenecks and low effective population size (Dlugosch et al., 2015; Dlugosch & Parker, 2008a, 2008b). Furthermore, the weak within‐population genetic substructure and large genetic neighbourhood sizes exhibited by the population support the “high connectivity” hypothesis, thereby contradicting the assumption that Tench has a low capacity for natural dispersal (Moyle, 1976). Contrary to what would be expected for a species expanding its geographic range across hundreds of kilometres, Tench suffered no substantial genetic diversity losses from repeated founder events.

### 4.1 Demographic changes and bottlenecks

We found evidence of a recent demographic bottleneck in the Tench population. Across eight scenarios representing demographic trajectories with and without population bottlenecks, DIYABC identified the group of scenarios incorporating a recent bottleneck as most likely. The most likely scenario included a bottleneck and no subsequent recovery, suggesting that the effective population size has not increased substantially following the recent demographic expansion. This uncoupling was further corroborated by the linkage disequilibrium estimate of a contemporary effective population size in the low hundreds. Small effective population size relative to the census population size (effective population size/census population size ratio < <0.5) is common in species with high fecundity and early life‐stage mortality (Bernos & Fraser, 2016; Palstra & Fraser, 2012), like Tench (Ablak Gürbüz, 2011). Positive internal relatedness values suggested inbreeding—another mechanism that can explain the small effective population size. Theoretically, an effective population size of >100 individuals is required to limit fitness losses due to inbreeding depression and >1000 individuals to retain evolutionary potential (Frankham et al., 2014). While this conclusion should be treated with caution as our analyses combined individuals born in different years, which can bias effective population size estimates downwards (Waples et al., 2014), Tench effective population size might be insufficiently large for the population to retain evolutionary potential in the long term.

Despite evidence for a drastic reduction in effective population size (<1.5% of the ancestral population size), Tench retained levels of genetic variability comparable to those exhibited by the species throughout the native range (Lo Presti et al., 2010) and native freshwater fishes in the invaded area (Brodeur & Bernatchez, 2018; Lujan et al., 2020). Although we detected significant temporal (~6 generations) changes in some metrics of genetic diversity at the individual (e.g. MLH, IR) and the population levels (Ho), the differences were generally modest. While we used permutation tests and computed metrics of genetic diversity insensitive to sample sizes to limit the consequences of uneven sample sizes, limited sample sizes for the historical samples hamper our conclusions. Regardless, this absence of reduced diversity likely results from rapid population expansion immediately following Tench introduction to Canada, highlighting the importance of population growth in mediating the severity of genetic diversity losses following bottlenecks (Kanuch et al., 2021; Zenger et al., 2003).

Consistent with previous studies, our results suggest that parameters inferred from DIYABC might bear some errors and downward biases, at least in point estimates (Cabrera & Palsbøll, 2017; Cammen et al., 2018). Most estimates had broad confidence intervals spanning most of the prior range. In particular, the prebottleneck population size was highly imprecise, with the upper bound of the range being more than 4‐fold greater than its lower bound. For estimated bottlenecked population size, the lower bound of the confidence interval (37 individuals) was remarkably close to our expectations, since Dumont et al. (2002) reported that about 30 live Tench were illegally imported to QC from Germany in the mid‐1980 s. On the other hand, the bottleneck was estimated to have occurred as little as 25 generations ago; however, based on an age at first maturity of 3 years old (Ablak Gürbüz, 2011), the Tench introduction to QC occurred only ~15 generations ago. Therefore, we cannot rule out the possibility that the bottleneck might have occurred before Tench introduction to eastern North America, perhaps reflecting the creation of a captive European source population. Further model evaluations are warranted to ensure that the approach can be applied reliably to obtain very specific insights (e.g. population size, timing of events), as opposed to general insights (e.g. occurrence of a bottleneck), from natural populations.

Regardless of whether the bottleneck occurred before or after the introduction, our analysis highlights that small introduced populations can do well in new environments despite bearing the genetic consequences of a bottleneck. Introduced populations flourishing in novel ecosystems are often cited as examples of the genetic paradox of invasions, which suggests that they are able to adapt successfully even after experiencing genetic bottlenecks (Allendorf & Lundquist, 2003). However, additional explanations to Tench success in the region should be considered (Estoup et al., 2016). First, analyses based on Tench habitat requirements previously identified the invaded region as suitable (Devaney et al., 2009; Kolar & Lodge, 2002). Therefore, long‐term adaptation to ecologically similar habitats in the native range might have facilitated the species' successful establishment in the invaded region (Bossdorf et al., 2008). Alternatively, Tench has a generalist life history, and, although the SLR might not perfectly match its habitat needs, the species may not need to become better suited to local environmental conditions to become invasive (Hufbauer et al., 2011). Second, the introduction was likely sourced from a fish farm or a site within proximity of human transportation systems. Adaptation to human‐altered habitats in the native range, potentially favoured by repeated introductions throughout the history of Tench (Clavero, 2019; Lajbner et al., 2011), could also be advantageous throughout the invaded region (Hufbauer et al., 2011), which contains two of Canada's largest cities (Montreal, Toronto) and is characterized by active farming and shipping industries. Third, genetic admixture could contribute to population fitness. Admixture between two distinct phylogroup has occurred in Tench populations throughout the native range without adverse fitness consequences (Karaiskou et al., 2020): as the eastern north‐American population contains both phylogroups (Lajbner et al., 2011), the benefits of admixture (e.g. greater genetic variation, masking of deleterious mutations) could have been revealed upon its introduction (Verhoeven et al., 2011). Consequently, further research on adaptive changes is required to discover whether the population truly is paradoxical (Estoup et al., 2016). Regardless, the introduced Tench population flourishes despite harbouring low genetic diversity and having gone through a recent bottleneck.

### 4.2 Range expansion and connectivity

The large genetic neighbourhood size and weak population substructure suggest that Tench is capable of extensive dispersal across the invaded range. For example, Tench sampled within a radius of 220 km were found to belong to the same breeding population, consistent with the average lifetime dispersal distance of 80 km and the maximum movement distance of 250 km inferred from otolith chemistry data (Morissette et al., 2021). Furthermore, the population exhibited population substructure suggesting limited dispersal capacity relative to the size of the landscape with weak genetic discontinuities throughout the invaded range. The genetic discontinuities did not coincide with major known geographic barriers (e.g. dams, rapids); instead, patches tended to be linked to larger waterbodies (e.g. lakes), where individuals might aggregate and/or be sampled in higher densities.

Although some species do exhibit reduced genetic diversity near the front of the range expansion compared to the core (Garroway et al., 2011; Watts et al., 2010), others found equivocal support for this theoretical prediction (Swaegers et al., 2013; Zenger et al., 2003). In Tench, there was a marginal loss of genetic diversity for some metrics, but genetic diversity was mostly preserved during the populations' geographic expansion. Simulation studies predict that, during range expansions, genetic diversity losses due to serial founder events can be mitigated by long‐distance dispersal and the genetic contributions from a large number of breeders (Miller et al., 2020; Williams et al., 2019). This was probably the case in our study as the large genetic neighbourhood size and lack of strong spatial structuring are consistent with high connectivity. In contrast, species with limited dispersal are more likely to resemble separate sub‐populations, which are more likely to lose genetic diversity through genetic drift due to reduced effective population sizes (Wright, 1946). Consequently, the size of the genetic neighbourhood relative to the area of range movement, which primarily reflects species' dispersal patterns, could be a useful predictor of the fate of genetic variation in populations undergoing range expansions.

Our results also highlight that range expansions can have different outcomes on spatial patterns of genetic diversity in populations expanding in multiple directions. We found a small, but significant, loss of genetic diversity for several metrics along the northern invasion axis (towards the SLR), while genetic diversity was preserved or increased along the southern Lake Champlain invasion axis. This result is compatible with two nonexclusive hypotheses related to neutral evolutionary processes. First, Tench dispersal characteristics might result in stochastic changes in allelic frequencies among invasion fronts. Indeed, fish movement is best described by dispersal kernels with greater likelihood of long‐distance dispersal events (Radinger & Wolter, 2013); this shape of dispersal can result in the random fixation of alleles, which might explain the observed differences between invasion fronts (Williams et al., 2019). Second, spatial environmental heterogeneity might influence genetic drift in such a way that it leads to variability among invasion fronts (Andrade‐Restrepo et al., 2019; Goodsman et al., 2014; Swaegers et al., 2013). For example, individuals in Lake Champlain might experience less pronounced genetic drift than those in the SLR because the homogeneous lacustrine habitat should facilitate multi‐directional dispersal and better water quality, due to lower agricultural and industrial footprint, which might sustain larger populations (Excoffier et al., 2009; Shirk et al., 2014).

### 4.3 Implications for Tench management

Our results cast doubt on the potential for complete eradication of Tench in the invaded region. To keep areas of conservation priority free of Tench, the large neighbourhood size indicates that eradicating the species within a ~112 km radius would be necessary to prevent recolonization. Even if this could be achieved, the area might eventually be recolonized as lifetime dispersal distances up to 250 km have been documented (Morissette et al., 2021). Consequently, instead of eradication, management plans should aim at managing the species to minimize its impacts across the invaded range. Control efforts should include initiating research to prevent Tench movement into the Great Lakes and to improve capture and control methods in high‐density areas, promoting awareness so fishers do not return captured Tench to the water and inadvertently transport it in their bait‐bucket, and improving collaborations between management agencies to build a common understanding of the problem and initiate structured decision‐making.

Second, our results confirm that Tench capacity for dispersal is higher than previously expected (Avlijas et al., 2018; Cudmore & Mandrak, 2011; Kolar & Lodge, 2002) and suggest that colonization of the Laurentian Great Lakes is imminent (Avlijas et al., 2018; Morissette et al., 2021), as perhaps foreshadowed by the capture of a live specimen in Lake Ontario in 2018, more than a genetic neighbourhood size ahead of the known invasion front. Because this individual did not differ genetically from the rest of the invaded range, its presence in Lake Ontario might be the result of a long‐distance dispersal event (natural or human‐aided). Alternatively, it could indicate the presence of a sleeper population (i.e. an established population persisting in low abundance: Spear et al., 2021) ahead of the known invasion front. This possibility warrants the implementation of targeted, cohesive monitoring efforts including both conventional and eDNA early detection approaches at the invasion front near the Laurentian Great Lakes. eDNA analysis, previously highlighted as an efficient tool to detect Tench DNA in the area (García‐Machado et al., 2021), might be especially useful to detect a sleeper population that might be below the detection threshold of conventional sampling gear (Spear et al., 2021). Targeted monitoring could enable the detection of, and rapid response to, the species when it is still at low abundance in the Great Lakes, where the species is predicted to flourish (Devaney et al., 2009).

### 4.4 Relationship to previous work

Prior work investigating the genetic consequences of species introductions in the Laurentian Great Lakes found high levels of genetic diversity and reduced founder effects in several invasive species, including Round Goby (*Neogobius melanostomis*) (Brown & Stepien, 2009; Snyder & Stepien, 2017), Zebra and Quagga mussels (*Dreissena polymorpha*, *D. bugensis*) (Stepien et al., 2002), Tubenose Goby (*Proterorhinus marmoratus*) (Stepien & Tumeo, 2006), and Ruffe (*Gymnocephalus cernua*) (Stepien et al., 2005). However, in these invasions, high propagule pressure and multiple introductions from distinct genetic sources explained the species' ability to retain high levels of genetic diversity throughout the invaded range. In our study, it is noteworthy that 30 individuals (<1.5% of the ancestral effective population size) followed by a rapid increase in population size maintained high levels of genetic diversity. Although the Tench in eastern North America exhibited the genetic signature of a bottleneck, it may be a remnant of bottlenecks that occurred prior to the introduction in the source population. This result is important for the conservation of small populations, as it suggests that severe reduction in historical population size does not always result in genetically depauperated populations.

Previous evidence from the Great Lakes suggested that losses in genetic diversity during range expansions might be a general characteristic of some populations (Brown & Stepien, 2009). For example, genetic diversity was lower in core areas compared to peripheral populations in Sea Lamprey (*Petromyzon marinus*) (Bryan et al., 2005); however, Round Goby retained high levels of genetic diversity in recently established peripheral populations, a likely consequence of stratified dispersal (Bronnenhuber et al., 2011). In contrast, we found some evidence for variability in the genetic consequences of range expansions across expanding fronts of the same invasive population. We believe that this difference might be explained by environmental differences between the southern and northern invasion front of our species and perhaps stochastic consequences of dispersal and neutral evolutionary forces (Excoffier et al., 2009; Miller et al., 2020).

### 4.5 Study caveats

Several issues might influence the results of our study. First, the Tench population is relatively new to the studied area, and it is possible that the effects of isolation‐by‐distance and the landscape context on population substructure require time to be realized (Anderson et al., 2010; Reding et al., 2013). However, it is worth noting that landscape structures influenced spatial genetic patterns very early (1–14 generations) in a simulation study, especially in highly vagile species (Landguth et al., 2010). Second, our methods do not allow inferences related to scenarios of multiple introductions from the same source population or from low‐differentiated populations. However, the strong pattern of isolation‐by‐distance highlighted by the sPCA suggests that, *if* multiple introductions from undifferentiated sources have occurred, the invasive population retained its original genetic signature. Third, conclusions related to differences in genetic diversity between the two time periods are hampered by the facts that the historical samples were limited in number and their DNA, despite meeting our quality threshold, more degraded than that of the contemporary samples. As a result, allelic dropout could have led to the underrepresentation of heterozygous genotypes in the historical samples, contributing to the observed differences between the two time periods Fourth, spatial patterns of genetic diversity and structure are simultaneously shaped by ongoing range expansion and gene flow, which are themselves influenced by landscape heterogeneity and dispersal. These processes operate on different spatial and temporal scales, and their respective influence on spatial patterns of genetic diversity is difficult to disentangle (Cushman, 2015). To confirm the results reported here, future research should employ simulation modelling implementing spatially explicit individual‐based simulation frameworks, such as CDMetaPOP (Landguth et al., 2017).

## 5 CONCLUSIONS

Understanding the consequences of founder effects and population bottlenecks for population persistence in new environments is of great practical interest, as these eco‐evolutionary challenges are commonly experienced by both invasive and endangered species (Colautti et al., 2017). We used a recently introduced invasive population as a model to examine the consequences of founder events and bottlenecks on patterns of genetic diversity and structure. This study provides an example of a small, isolated vertebrate population that proliferated in a new environment despite reduced effective population size and a recent bottleneck (cf. Dlugosch & Parker, 2008a, 2008b; Uller & Leimu, 2011). Furthermore, the population did not exhibit a consistent decay in genetic diversity from the invasion core to the margins, despite the large size and habitat diversity of the invaded range. How this will impact adaptation (Excoffier et al., 2009) and dispersal (Cobben et al., 2015) as the population continues to expand into new habitats remains to be discovered. Notably, theoretical predictions suggest that, if dispersal is high enough, populations relatively well adapted to the introduced range can rapidly spread into the entire habitable range (Andrade‐Restrepo et al., 2019). Range expansion itself could provide an opportunity for phenotypic changes to occur via spatial sorting, the evolution of dispersal‐enhancing traits due to the concentration of fast‐dispersers at the expanding front (Shine et al., 2011). However, our study also shows that, in natural settings, populations spreading in multiple directions within a single range expansion might exhibit different evolutionary trajectories. A better understanding of factors generating variability in the genetic outcomes of range expansions could allow us to make more accurate predictions related to evolution during range expansions, whether in response to introduction to a new range or to track suitable habitat conditions.

## CONFLICT OF INTEREST

The authors declare no conflicts of interest.

## BENEFIT‐SHARING STATEMENT

A research collaboration was developed between all collaborators, including scientists in academic and government agencies. Results of the research were shared with the broader scientific community. The research addresses an important topic for conservation, the rapid spread of an invasive species of major concern for native species in the St Lawrence River.

## Supporting information


Figures S1–S4

## Data Availability

Individual genotype data and associated metadata was made available on NCBI (https://www.ncbi.nlm.nih.gov/sra/PRJNA903386). Code was uploaded to github (Linkr1_TincaTinca_Quebec).
