# Supplementary material for: Genetic diversity and structure of a recent fish invasion: Tench (Tinca tinca) in eastern North America
Source: Evol Appl. 2022 Dec 20;16(1):173–88. doi: 10.1111/eva.13520 (PMC9850014; doi:10.1111/eva.13520)
Supplement: Supplementary file 1 — Figures S1–S4 [file EVA-16-173-s001.docx]

Figure S1: Investigation of the effect of the genetic distance allowed between two stacks (M) and the number of mismatches in the catalog (n) in de-novo stacks assembly for Tench (*Tinca tinca*) in eastern North America on: a) the number of assembled loci present in >70% of the samples, b) the number of polymorphic loci, c) the number of SNPs, d) the number of SNPs per loci, and e) the proportion of potentially erroneous SNPs. For a given n, we observed that the number of polymorphic loci was the highest for M2 and M3 and the number of SNPs for M3. However, with M3, we observed a greater proportion of potentially erroneous SNPs and noted that there were more loci with high numbers of SNPs, suggesting that some loci might erroneously merge together for higher values of M. For a given M, the number of polymorphic loci and SNPs were greater for n=M-1; however, this parameter value was also associated with greater proportions of potentially erroneous SNPs and loci with higher numbers of SNPs. Given that we expect fixed differences to be rare in our dataset, we chose to select n=M. Accordingly, we identified M2 and n2 as the optimal parameters for our dataset.


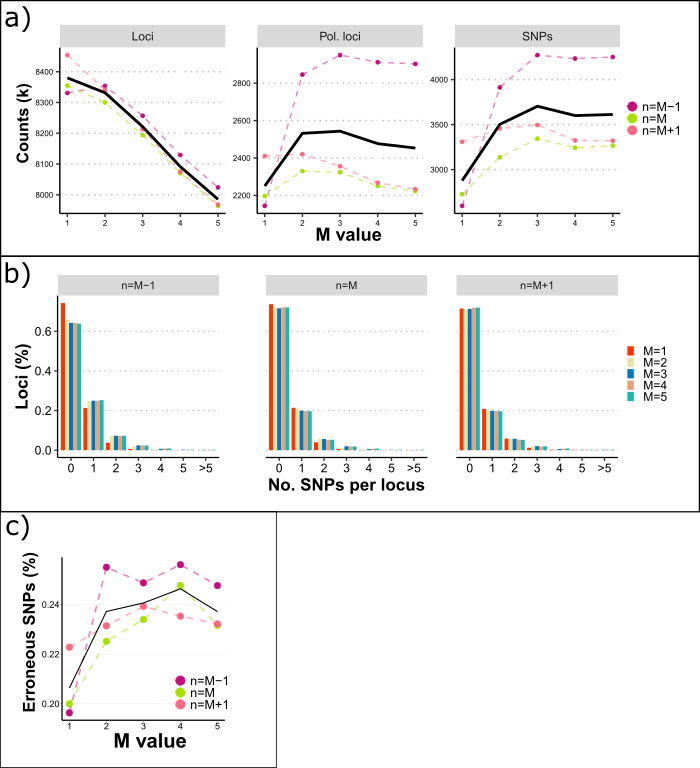


Fig. S2: Variation in effective population size estimates from the linkage disequilibrium method (Ne_LD_) as a function of excluding rare alleles (P_crit_) for Tench (*Tinca tinca*) in eastern North America. Point estimates and associated 95% jacknife confidence intervals are shown.


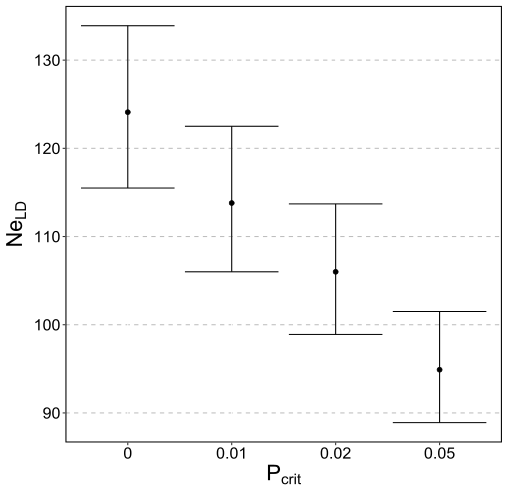


Fig S3: Randomization tests histogram for internal relatedness (IR) and multilocus heterozygosity (MLH) for differences between the historical and contemporary invasive population of Tench in eastern North America. Red lines indicate the range within 95% of the values fall, and the dotted black line indicate the observed mean difference between the two populations.


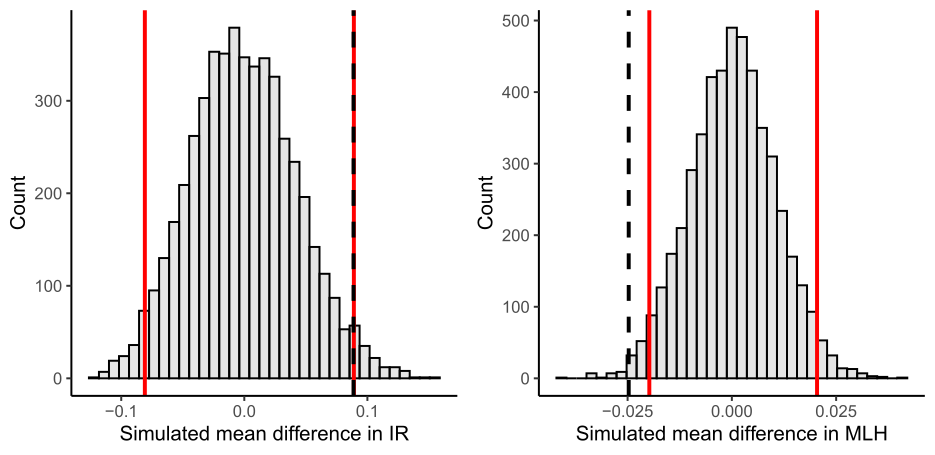


Fig S4: Projection of the observed Tench data on a single LDA axis for the group-level analysis (upper figure) and two LDA axes for the scenario-level analysis (lower figure). The two groups represent scenarios without (group of scenarios 1, scenario 1:4) and with bottleneck (group of scenarios 2, scenario 5:6).

| **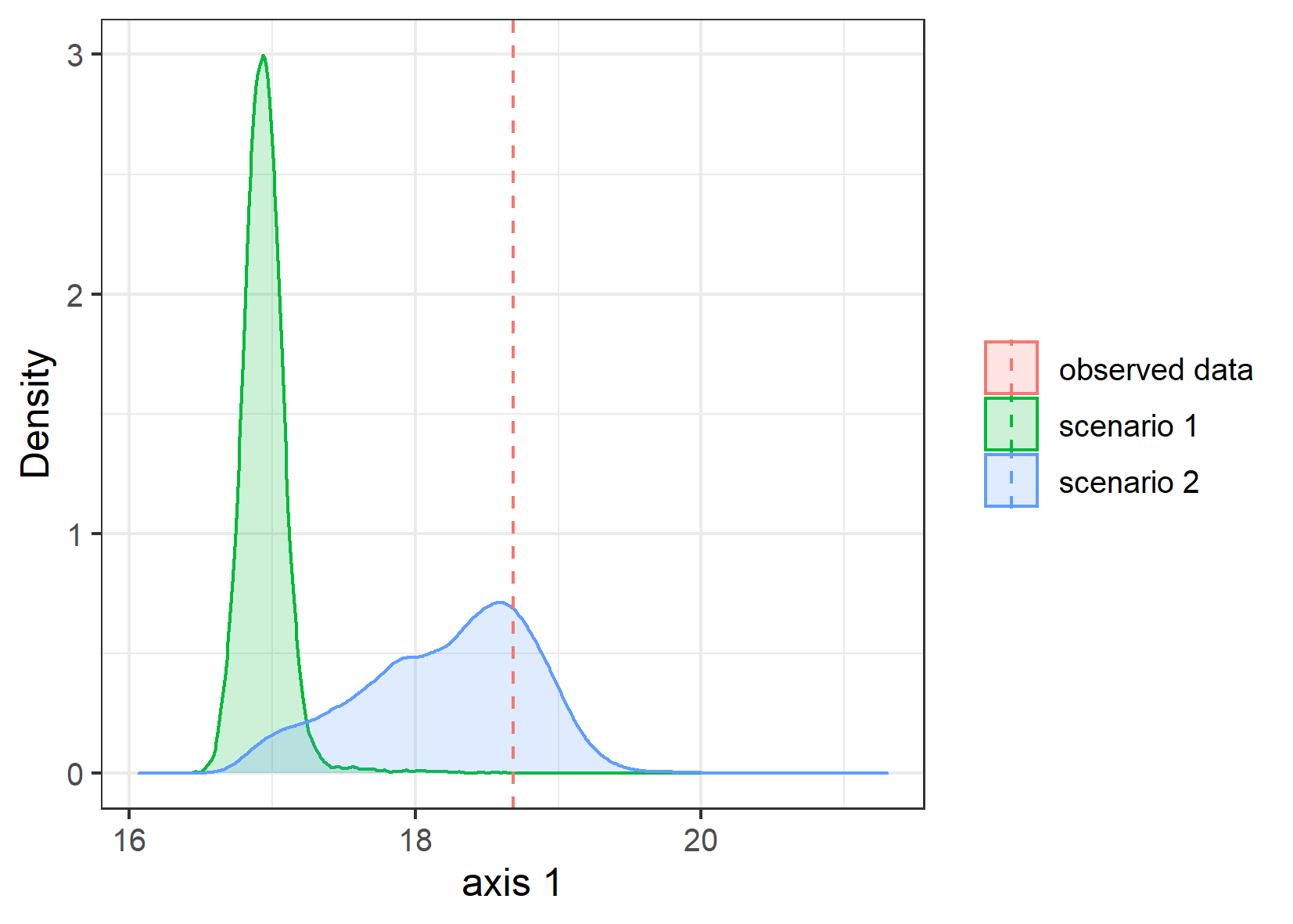** |
| --- |
| 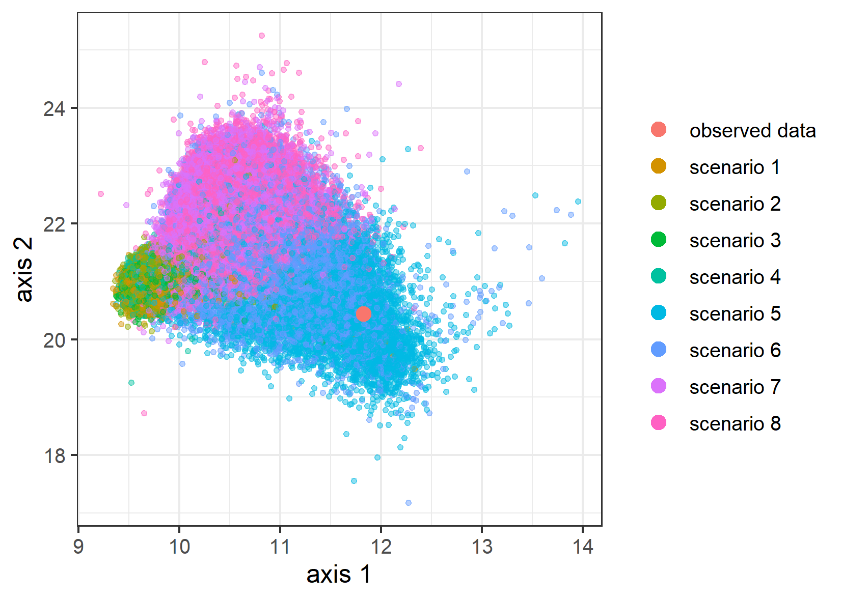 |
